# Treatment of large plasma volumes using specific immunoadsorption to desensitize ABO-incompatible kidney-transplant candidates

**DOI:** 10.15171/jnp.2016.17

**Published:** 2016-06-29

**Authors:** Lionel Rostaing, Asma Allal, Arnaud del Bello, Federico Sallusto, Laure Esposito, Nicolas Doumerc, Bénédicte Debiol, Audrey Delas, Xavier Game, Nassim Kamar

**Affiliations:** ^1^Department of Nephrology and Organ Transplantation, CHU Rangueil, Toulouse, France; ^2^INSERM U563, IFR–BMT, CHU Purpan, Toulouse, France; ^3^Université Toulouse III Paul Sabatier, Toulouse, France; ^4^Department of Urology, Andrology, and Transplantation, CHU Rangueil, Toulouse, France; ^5^Etablissement Français du Sang de Midi-Pyrénées, CHU Purpan, Toulouse, France; ^6^Laboratory of Histopathology, CHU Rangueil, Toulouse, France; ^7^INSERM U1043, IFR-BMT, CHU Purpan, Toulouse, France

**Keywords:** ABO-incompatible kidney transplantation, Specific immunoadsorption, Glycosorb®, Large plasma volumes

## Abstract

**Background:**

ABO-incompatible (ABOi) kidney-transplantation has very good long-term results, i.e. similar to those observed for living-kidney ABO-compatible transplantation. This is because patients are desensitized at pretransplant using apheresis and rituximab therapy, with tacrolimus-based immunosuppression.

**Objectives:**

To assess the efficacy of a single, pretransplant (Day –1), specific immunoadsorption session using Glycosorb® columns (anti-A or anti-B; Glycorex Sweden) to treat large volumes of plasma (up to 18 L).

**Patients and Methods:**

Prospective single-center study evaluating 12 consecutive patients (6 males), aged 40 (23–59) years. Incompatibilities were A into 0 (8), B into 0 (3), and AB into 0 (1). Pretransplant desensitization relied on rituximab (D–30), tacrolimus, mycophenolic acid, and steroids (all started on D–13), and a single session of specific immunoadsorption on D–1. Immunoadsorption was coupled in tandem with a hemodialysis session.

**Results:**

Overall, 15 L (11–18) of plasma were treated per patient, i.e., 0.2 (0.11–0.36 L/kg). Isoagglutinin titers were 1/16 (1/5–1/64) before the procedure, decreasing after 6 hours to 1/5 (1/1–1/16 *P* = 0.008), and to 1/2 (1/1–1/8; *P* = 0.05) at completion of the session. The next day, i.e., the day of transplantation, there was no rebound of isoagglutinins [1/4 (1/1–1/5); *P* = ns]. The procedure was well tolerated with no side-effects and no significant changes in hemoglobin level, platelet counts, fibrinogen, or albumin levels.

**Conclusions:**

For ABOi kidney-transplantation, a single, longer, specific immunoadsorption session was very efficient at 1-day pre-transplantation with no rebound. These results should be confirmed when isoagglutinin titers are higher (≥120).

**Implication for health policy/practice/research/medical education:**

More and more end-stage renal disease-patients require a kidney transplant; due to a lack of deceased donor, living-kidneytransplantation is a good option. However, sometimes we face ABO incompatibility (ABOi). ABOi kidney transplantationis associated with very good long-term results provided pretransplant desensitization that relies on T-cell-targetedimmunosuppression and apheresis. The most specific and efficient apheresis is specific immunoadsorption with Glycosorb®columns.However, the price is around 3000 euro per column. Each patient requires a few sessionprior to transplantation. In this study we demonstrate that using a single specific immunoadsorption column with treating largeplasma volumes (up to 18L) is very efficient, is associated with no isoagglutinin rebound and thereby might reduce dramaticallythe cost of ABOi desensitization.

## 1. Background

Worldwide, many people suffer from end-stage renal disease (ESRD), of which about a third are eligible for kidney transplantation. Because there is a scarcity of deceased kidney donors, physicians have developed live-kidney donation: however, in doing so we often face ABO-incompatibility (ABOi). In this setting, kidney transplantation is possible provided pre-transplant desensitization is conducted to decrease isoagglutinin titers below a certain threshold at pretransplant. Isoagglutinins, when present at high titers, can cause acute antibody-mediated rejection (1-4).

Pretransplant desensitization is based on two combined approaches (5-9). The first is immunosuppression, based on steroids, tacrolimus, and mycophenolic acid, plus rituximab. Treatment is started at ~10 days pre-transplant and leads to inhibition of isoagglutinin re-synthesis. The second approach approach is apheresis, which removes isoagglutinins. Apheresis can be based on plasmapheresis (PP) or double-filtration plasmapheresis (DFPP); although effective PP associated with removal of clotting factors, which can cause serious bleeds during the peri-transplantation period (10-12). An elegant method to efficient eliminate isoagglutinins and to preserve clotting factors is to perform specific immunoadsorption, which can deplete either anti-A or anti-B isoagglutinins. The Glycosorb®-ABO columns are based on carbohydrates (blood group antigens) (anti-A or anti-B Glycosorb® columns); however, Glycosorb® columns (Glycorex, Sweden) are not reusable and are very expensive, i.e., ~3000 euros each (13,14).

In order to achieve efficient desensitization at pretransplant, we need to use one to four columns according to the isoagglutinin titer: this is a huge economic burden. Each immunoadsorption session can treat around two plasma volumes. After the first immunoadsorption session there is often isoagglutinin rebound. In the present study, we hypothesized that treating high plasma volumes (≈15 to 20 L per session) within a single session could be efficient with regards to isoagglutinin titers and would also not cause isoagglutinin rebound. This would result in a large decrease in the number of columns needed per patient. In order to demonstrate this, we included 12 patients whose isoagglutinin titers were not too high prior to desensitization, i.e., lower than 1/64.

## 2. Objectives

Of that, study were to assess whether treating high plasma volumes (15 to 20 L per immunoadsorption session) within a single session could be efficient with regards to isoagglutinin titers and would also not be associated with isoagglutinin rebound.

## 3. Patients and Methods

### 3.1. Study population

This prospective single-center study included all consecutive ABO-incompatible (ABOi) kidney-transplant recipients for whom living-kidney transplantation was scheduled. Their pre-desensitization isoagglutinin titers had to be between >1/8 and ≤1/64. At 30 days pretransplant, they were infused with rituximab (375 mg/m²). At 13 days pretransplant, T-cell-targeted immunosuppression was started, which included tacrolimus at 0.2 mg/kg/d (targeting trough levels of 8–10 ng/mL), mycophenolic acid (Myfortic® 360 mg b.i.d.), and prednisolone (0.5 mg/kg/d). We planned a long immunoadsorption session, i.e.,7–9 hours, for all 12 patients on the day prior to transplantation using specific anti-A or anti-B Glycosorb® columns. The immunoadsorption session was always coupled in tandem with a hemodialysis session, as previously described by Maggioni et al (15).

Briefly, the session was started at 8 am when we first set up the immunoadsorption devices. The plasma was separated using a Comtec® machine (Fresenius, Germany) at a rate of 60 mL/min; the plasma was then passed through the Glycosorb® column and then reinfused back into the patient. Using the same Y-shaped needle, the other arterial branch was used to connect the patient to the hemodialysis generator; the hemodialysis session was scheduled for 4 hours. During the immunoadsorption session, at 2.5/3-hour intervals, we assessed isoagglutinin titers to ensure that they were steadily decreasing. Overall, the immunoadsorption session lasted 7–9 hours and treated 15–20 L of plasma. However, if at 6 hours after starting immunoadsorption the isoagglutinin titer was already below 1/8, we stopped the session at 7 hours.

Isoagglutinins were titrated using an in-house hemagglutination technique. Donor-specific alloantibodies (DSAs) were detected by Luminex® assays using the LABScreen Single Antigen kit (One Lambda, US) according to the manufacturer’s instructions.

### 3. 2. Ethical issues

The research followed the tenets of the Declaration of Helsinki. Also, this project was approved by ethics committee of Toulouse University Hospital, France.

### 3. 3. Statistical analysis

Reported values are the means and standard deviations or the medians (ranges), as appropriate. The two groups of data were compared using the chi-square test or Fisher’s exact test for qualitative data. Additionally, Wilcoxon’s test or student’s* t* test for quantitative data was used. A *P* value of <0.05 was considered statistically significant.

## 4. Results

We included 12 patients (6 females) who had a median age of 42 (range: 23–59) years. All but three were receiving hemodialysis therapy. In most cases (11 cases), it was a first kidney transplant. Only one patient (no. 2) had donor-specific alloantibodies. ABOi was A into O in eight cases, B into O in three cases, and AB into O in one case (Table 1).

**Table 1:** Demographics of the study population

**Patient**	**Gender** **(F/M)**	**Age (years)**	**Date of transplantation (dd/mm/yy)**	**Time on dialysis (months)**	**Cause of ESRD**	**Rank of KTx (n)**	**Blood group**		**DSA (Y/N)**
							**Donor**	**Recipient**	
1	F	55	11/03/15	35	Type 1 diabetes	1^st^	A	O	N
2	F	38	08/04/15	171	Renal hypoplasia	2^nd^	A	O	Y
3	F	31	22/04/15	29	Barter syndrome	1^st^	A	O	N
4	M	23	05/08/15	Preemptive	Renal hypoplasia	1^st^	A	O	N
5	M	42	26/08/15	68	Nephroangiosclerosis	1^st^	A	B	N
6	M	59	25/09/15	8	Nephroangiosclerosis	1^st^	B	O	N
7	F	48	30/09/15	Preemptive	Lupus nephritis	1^st^	B	O	N
8	F	35	14/10/15	93	Type-1 diabetes	1^st^	AB	O	N
9	F	58	28/10/15	26	FSGS	1^st^	A	O	N
10	M	42	04/11/15	Preemptive	IgA nephropathy	1^st^	A	O	N
11	M	33	25/11/15	10	IgA nephropathy	1^st^	B	O	N
12	M	35	09/12/15	42	GNC	1^st^	A	O	N

Abbreviations: M, male; F, female; ESRD, end-stage renal disease; KTx, kidney transplantation; DSA donor-specific alloantibodies; Y, yes; N, no.

The baseline isoagglutinin titer, i.e., before rituximab infection, was 1/16 (range: 1/5–1/64). Four weeks after rituximab infusion, i.e., at one day pre-transplantation, isoagglutinin titers were 1/16 (range: 1/5–1/64; *P*=ns). Six hours after starting immunoadsorption, isoagglutinin titer had significantly decreased to 1/5 (range: 1/1–1/16; *P*=0.008); at the end of the session, i.e., at 8–9 hours after starting immunoadsorption, isoagglutinins titers were significantly further reduced from 1/5 (range: 1/1–1/16) down to 1/2 (range: 1/1–1/8; *P*=0.05). After the session there was no rebound; the next morning, the isoagglutinin titer was 1/4 (range: 1/1–1/5; *P*=0.6). After transplantation, isoagglutinin titers remained stable at 1/2 (range: 1/1–1/8) on day 1 and 1/2 (range: 1/1–1/10) on day 5. Overall, 15 L (range; 11–18) of plasma were treated per patient, i.e., 0.2 (range: 0.11–0.36 L/kg) (Table 2).

**Table 2:** Outcomes of isoagglutinin titers after the specific IA session

**Patient**		**Specific isoagglutinin titers before and after the IA session**													**Volume of plasma (L) (L/BW)**	
		**1 month pre-KTx**				**Day -1**				**Day 0**	**Day 1**		**Day 5**			
				**H0**		**H6**		**End of session**								
1		1/5		1/5		1		1		1/2	1/2		1/2		14 (0.18)	
2		1/64		1/8		ND		1/5		1/5	1/5		1/10		11 (0.11)	
3		1/32		1/30		1/5		1/2		1/5	1/2		1		16 (0.26)	
4		1/16		1/20		1/5		1/2		1/5	1/2		1/2		18 (0.36)	
5		1/10		1/10		1/5		1		1	1/5		1/2		15 (0.13)	
6		1/16		1/64		1/5		1/2		1/5	1/8		1/2		15 (0.25)	
7		1/32		1/32		1/5		15		1/5	1/2		1		18 (0.29)	
8		1/16	1/5	1/32	1/16	1/5	1/5	1	1/5	ND	1/4	1	1/2	1	17 (0.2)	
9		1/10		1/10		1/4		1		1	1		1/2		15 (0.2)	
10		1/16		1/16		1/5		1/2		1	1		1		16 (0.19)	
11		1/10		1/8		1/4		1/4		1/2	1/2		1		14 (0.2)	
12		1/32		1/16		1/16		1/8		1/4	1/4		1/2		18 (0.21)	

Abbreviations: IA, immunoadsorption; BW, body weight (expressed in kg); H0, at the time when IA was started; H6, 6 hours after starting IA; KTx, kidney transplantation. Statistical analyses (Isoagglutinin titers): 1 month pre-kidney transplant (KTx) vs. H0: *P*=ns; H0 vs. H6: *P*=0.008; H0 vs. end of session: *P*=0.003; H6 vs. end of session: *P*=0.05; end of session vs. day 0: *P*=; D0 vs. D1/D0 vs. D5; D1 vs. D5: *P*=ns.

### 4.1. Tolerability to the immunoadsorption session

Before and after the immunoadsorption session, we monitored hemoglobin, fibrinogen, and albumin plasma levels, as well as platelet counts. None of these parameters were significantly affected by the technique (Table 3). During and after the transplant procedure, there was no significant bleeding and the need for transfusion during or immediately after surgery was average.

**Table 3:** Biochemical and hematological parameters before and after the IA session

**Patient**	**Hemoglobin (g/dL)**		**Platelets (x 10** ^3^ **/mm** ^3^ **)**		**Fibrinogen (g/L)**		**Albumin (g/L)**		**IA plus HD**
	**Pre-**	**Post-**	**Pre-**	**Post-**	**Pre-**	**Post-**	**Pre-**	**Post-**	
1	10.3	9.7	214	176	4.2	3.6	30	37	Y
2	8.5	8.2	165	119	2.8	2.5	36	33	Y
3	11.5	10.8	196	148	2.4	2.4	33	32	Y
4	8.2	8	329	322	ND	2.5	41	39	Y
5	10.1	10.8	159	178	2.3	2.6	37	40	Y
6	13.2	11.4	181	162	3.4	2.8	37	29	N
7	9.8	8.9	225	195	2.7	2.6	34	31	Y
8	9.8	10	384	343	2.8	2.6	39	37	Y
9	11	10.4	341	286	2.7	2.5	36	33	Y
10	11.4	11.2	222	211	2.7	2.6	ND	ND	Y
11	12.1	10.8	177	156	2.6	2.4	ND	ND	Y
12	13.4	12.3	193	165	ND	ND	42	38	Y

Abbreviations: IA, immunoadsorption; Y, yes; N, no; HD, hemodialysis Statistical analyses: For each variable there was no significant difference between pre- and post-IA sessions.

### 4.2. Patients’ outcomes

The median follow-up after transplantation was 80 (range: 15–280) days. Five patients (41%) presented with delayed graft function defined by having a serum-creatinine level of >400 µmol/L on post-op day 5. In fact, these five patients presented with acute antibody-mediated rejection within the immediate post-transplant period, i.e., before post-op day 5, despite having very low isoagglutinin titers: this occurred as1/1 in two cases and 1/2 in three cases. None of these patients had any pretransplant DSAs. At the time of the acute rejection episode (and at one month later) we checked for DSAs: none were present in the five patients. The five cases were successfully treated with pulses of methylprednisolone (10 mg/kg for 3 consecutive days) (n=5) and PP sessions (n=3). Only two patients also received two infusions of rituximab. All five patients recovered (Table 4).

**Table 4:** Post-transplant outcomes

**Patient**	**Serum creatinine (µmol/L)**						**Rejection (Y/N)**	**Time of rejection (days)**	**Type of rejection**
	**Time since KTx (days)**	**D0**	**D1**	**D5**	**D30**	D90*			
1	280	454	249	66	74	97	N	NA	NA
2	255	451	547	228	151	116	N	NA	NA
3	245	424	283	98	119	132	N	NA	NA
4	135	530	568	482	223	224	Y	D5	AAMR
5	115	991	942	733	203	161	Y	D5	AAMR
6	85	842	543	134	145	110	N	NA	NA
7	80	510	304	176	86	110	N	NA	NA
8	65	297	383	629	113	90	Y	D3	AAMR
9	55	579	578	173	86	90*	N	NA	NA
10	50	570	475	160	158	148	N	NA	NA
11	30	787	545	624	132	143*	Y	D4	AAMR
12	15	814	737	985	195	177 *	Y	D5	AAMR

Abbreviations: KTx, kidney transplantation; D, day; Y, yes; N, no; *, or last follow-up; AAMR, acute antibody-mediated rejection. Statistical analyses (serum creatinine): D0 vs. D1: *P*=ns; D0 vs. D5: *P*=0.042; D1 vs. D5: *P*=ns.

## 5. Discussion

To the best of our knowledge, this is the first prospective study to report treating very large plasma volumes in a single, pretransplant, specific immunoadsorption session to desensitize ABO kidney-transplant candidates.

ABOi kidney transplantation is associated with very good long-term results, i.e., similar to those observed with ABO-compatible live-kidney transplantation. These outcomes have been reported not only from Japan, where this type of transplantation was largely developed (16), but also from Europe (17) and the United States (18). However, to make ABOi transplantation successful, we need to perform desensitization at pre-transplant so that, after transplantation, accommodation could take place within a few weeks (19,20). This should occur despite ABOi and despite C4d deposition within the allograft (a hallmark of complement activation), and renal function should remain normal. However, in some cases, within the first few posttransplant weeks, antibody-mediated acute rejection can occur, despite having low titers of isoagglutinins. In these cases, rejection can cause impaired allograft function (21).

At pretransplant, desensitization relies on immunosuppression that targets both T and B cells, and apheresis to remove isoagglutinins. Apheresis techniques in the United States are based on PP because other techniques have not yet been registered; in Japan they are based on DFPP. Both techniques are effective at removing isoagglutinins, but the drawback is that both methods also remove other factors, such as clotting factors, including fibrinogen and factor XIII, which can then cause peri-operative bleeds, especially if the last session was performed on the pre-transplant day (10).

In Europe, in addition to these two techniques, we have the possibility of using specific immunoadsorption, which relies on protein-A columns covered with anti-A or anti-B antibodies. These columns are non-reusable, even though a recent study has shown that it is technically feasible to re-use them and that they remain efficient even after a few reuses (22). However, the price is around 3000 euro per column. The advantage of specific immunoadsorption over other techniques (PP, DFPP) is that it is specific to the culprit antibody and does not remove clotting factors. In addition, a study has shown that these specific columns can also remove complement factors, such as C1q, C3, or C4 fractions: this may have beneficial effects, especially when ABOi is associated with HLA incompatibility (23). However, because some patients require more than one specific immunoadsorption session at pre-transplantation, the extra cost of desensitization using this technique is very high compared to DFPP, where the only cost is the filter, and to PP, where the only cost is replacement fluid (albumin or fresh frozen plasma). Thus, the wide-spread use of specific immunoadsorption has been compromised until costs can be reduced, even though this technique is more effective than the other two methods with regards specificity.

We postulated that we could use a single-use specific immunoadsorption session over a longer period to treat greater plasma volumes and still preserve efficacy and avoid a rebound of isoagglutinins the next day. Because treating larger volumes of plasma extends the sessions to 7–9 hours, we decided to combine, in tandem, specific immunoadsorption and hemodialysis, as has been recently reported (15). We also decided to perform this tandem procedure on the day before transplantation in order to achieve low isoagglutinin titers and good biochemical parameters on the day of transplantation.

For this proof-of-concept, we decided to only include patients whose isoagglutinin titers at pre-desensitization were <1/120. Our routine desensitization protocol included rituximab infusion on day 30 pretransplant; however, this did not significantly decrease isoagglutinin titers, as measured 4 weeks later, i.e., on the day before transplantation. Conversely, a study from Sweden reported that a single rituximab infusion (375 mg/m^2^) without apheresis could significantly reduce isoagglutinins titers, even though very few patients achieved the target required to safely undergo the transplantation procedure. Of note, in our study, on the day prior to kidney transplantation and before immunoadsorption, the isoagglutinin titers were quite low, i.e.,1/16 (range: 1/5–1/64). This relatively low titer may have made it easier to further decrease isoagglutinin titers. Although we cannot extrapolate these results to those where isoagglutinin titers were 1/128 or greater prior to transplantation, at the end of our single, large plasma-volume treatment, the median isoagglutinin titer had significantly decreased by four-fold to 1/2 (range: 1/1–1/8). Moreover, the longer length of the session allowed immunoglobulins to refill from the extra vascular compartment into the intravascular compartment during the immunoadsorption session: thus, there was no rebound the following day, i.e., on the day of transplantation isoagglutinin titer was 1/4 (range: 1/1–1/5).

The lengthy procedure was very well tolerated; in order to improve the patients’ comfort we performed immunoadsorption and hemodialysis at the same time, as described previously by our team (15). This approach also saves nursing time, i.e., hemodialysis does not need to be performed after completing immunoadsorption.

We carefully monitored important parameters, such as hemoglobin level, platelet counts, fibrinogen, and albumin levels, at before and after immunoadsorption: none were significantly altered by the procedure. This is very reassuring. In addition, there were no significant bleeds or a need for large red blood-cell transfusion during or immediately after surgery. This contrasts with that reported in the setting of ABOi kidney transplantation, where pretransplant patients that undergo either PP or DFPP often have significant bleeding complications during and after surgery.

In our small series, five of the 12 patients experienced a very early posttransplant acute-rejection episode and had antibody-mediated features on a kidney biopsy. These rejections, although of abrupt onset, were not associated with increased isoagglutinin titers and were easily reversed with pulses of methyl prednisolone and PP. Only two patients also received rituximab therapy. It is well known that, in the setting of ABOi kidney transplantation, early post-transplant acute rejection is mediated by isoagglutinins even though the periphery titer may be low, i.e., there is no correlation between isoagglutinin titers and acute antibody-mediated rejection. Soon after transplantation, we also checked for donor-specific alloantibodies, which were not present in any of our patients.

## 6. Conclusions

We conclude from this single-center prospective study that, for ABOi kidney transplantation, a single, longer, specific immunoadsorption session was very efficient when given at one-day pre-transplantation, i.e., it decreased isoagglutinin titers by four-fold. In addition, there was no rebound on the day after transplantation. Moreover, this procedure was very well tolerated, particularly regarding hematological parameters. However, these results should be confirmed when isoagglutinin titers are higher (≥120).

## Limitations of the study

This is a single-center non-randomized study with a limited number of patients.

## Acknowledgments

We deeply acknowledge all the nurse team as well as the head nurse Mr Sébastion Maggioni from our apheresis and acute hemodialysis facility, without whom this study could not have been performed.

## Authors’ contribution

LR, AA, and NK designed the study and participated in writing the paper; ADB, LE, LR, and NK recruited the patients; FS, ND and XG performed the surgery; BD performed isoagglutinin assessments; AD analyzed the kidney allograft biopsies.

## Conflicts of interest

The authors declared no competing interests.

## Funding/Support

The authors declare that they have no competing financial interests in relation to the work described. This research was supported by Direction de la recherché Clinique (AOL) from Toulouse University Hospital, France.
